# Factors affecting response to furosemide stress test among critically ill hypoalbuminemic patients with AKI without prior albumin infusion

**DOI:** 10.1186/s12882-025-04532-2

**Published:** 2025-11-25

**Authors:** Amin Roshdy Soliman, Ahmed Yousry, Hoda Abdelhamid Maamoun

**Affiliations:** 1 Internal Medicine and Nephrology Department, Kasr Alainy Faculty of Medicine, Cairo University, Cairo, Egypt; 2 Cardiology Department, Zagazig University, Zagazig, Egypt

**Keywords:** Acute kidney injury, Hypoalbuminemia, Furosemide stress test

## Abstract

**Background:**

Acute kidney injury (AKI) is a common and serious condition often associated with hypoalbuminemia, which can influence the pharmacokinetics and efficacy of diuretics like furosemide. In critically ill patients, sepsis is the major cause of AKI, accounting for nearly 50% of cases.

**Objective:**

To evaluate whether AKI patients with hypoalbuminemia can respond to FST without albumin supplementation.

**Methods:**

This is a prospective quasi-experimental study. Patients were obtained from the intensive care unit of Cairo University Hospital with AKI stages 1 and 2 with hypoalbuminemia. A bolus of furosemide was administered at a dose calculated to be 1-1.5 mg/kg in a single dose to patients without a prior diagnosis of kidney disease and clinical signs of hypovolemia.

**Results:**

A total of 41 critically ill patients with AKI were enrolled, aged between 18 and 80 years, of whom 56.10% had diabetes mellitus, 53.70% were on at least one nephrotoxic medication, and 56.10% had sepsis as the cause of AKI. The median (IQR) albumin level was 1.9 g/dL (1.4–2.7). Among 41 hypoalbuminemic AKI patients included, 80.50% responded to FST without prior albumin infusion. Non-responders had significantly lower baseline serum albumin levels, median (IQR) 1(1–2) vs. 2 (1–3) g/dL, *p* < 0.002).

**Conclusion:**

AKI patients with mild-to-moderate hypoalbuminemia may still respond to FST without albumin infusion, although response rates decline with the increasing severity of hypoalbuminemia. The FST remains a valuable predictive tool in hypoalbuminemic AKI patients but warrants further investigation to optimize its utility in this population.

**Clinical trial number:**

Not applicable.

## Introduction

Acute Kidney Injury (AKI) is a common and serious complication that is associated with several adverse outcomes including death, need for renal replacement therapy (RRT), increased length of hospital stay, chronic kidney disease, and rising health care costs. Even mild forms of kidney injury influence short- and long-term morbidity and mortality through a progression of chronic kidney disease (CKD) and cardiovascular disease. Specific treatments for AKI are currently lacking and supportive care is the mainstay of therapy. Prevention is therefore of the utmost importance and relies on the identification of individuals at high risk for development of AKI early in the intensive care unit (ICU) admission [1–3].

In the critically ill, sepsis is the major cause of AKI, accounting for nearly 50% of cases so Suspicion of infection was made using the Sequential Organ Failure Assessment (SOFA) score. It is a scoring system that assesses the performance of several organ systems in the body (neurologic, blood, liver, kidney, and blood pressure/hemodynamics) and assigns a score based on the data obtained in each category [4, 5].

Critically ill patients with AKI may receive an exogenous source of vasopressin or its analog that increases sodium and water retention. These mechanisms might have the effect of amplifying the defect in water excretion in edematous AKI patients [6].

Since acute tubular injury is the most common cause of intrinsic acute kidney injury (AKI), a functional assessment focusing on renal tubular function was developed. Furosemide, a loop diuretic, exhibits pharmacokinetic properties that render it a useful functional tool in this context. Furosemide-induced increases in urine output may serve as a method to assess renal tubular integrity in the early stages of AKI. It is hypothesized that the kidney’s response, or lack thereof, to a furosemide challenge can act as a clinical assessment of tubular function and may help identify patients with severe tubular injury before the manifestation of overt clinical signs [7, 8].

The action and efficacy of furosemide differ considerably in patients with AKI. Several factors account for these differences as nephrotoxic medications, vasopressors, sepsis, and hypoalbuminemia [9].

Critically ill patients commonly exhibit moderate to severe hypoalbuminemia that can have a considerable effect on the pharmacokinetics and pharmacodynamics of drugs such as diuretics, furosemide, of which approximately 95% binds to plasma proteins [10]. Despite the universal use of the Furosemide Stress Test (FST) to assess renal tubular function in acute kidney injury (AKI), the influence of hypoalbuminemia and other clinical conditions, including nephrotoxic agents, vasopressors, diabetes, and sepsis, on the effectiveness of FST is still undetermined. This study aims to evaluate the effectiveness of the FST in stage 1 and stage 2 AKI patients and critically ill patients, and investigate the effect of the variables on the furosemide response. The outcome is intended to overcome the existing knowledge deficiency and enhance clinical understanding of renal function measurement in a high-risk population.

## Materials and methods

### Patient recruitment and screening

Patients were recruited from the Intensive Care Unit in Kasr Alaini Hospital. Patients who satisfied the following criteria were assessed for FST.

1. Meeting the AKI diagnostic criteria for Kidney Disease Improving Global Outcomes (KDIGO) guidelines, stage 1 and 2); Stage 1: Serum creatinine 1.5–1.9 times baseline or ≥ 0.3 mg/dL increase, Urine output < 0.5 mL/kg/h for 6 h, Stage 2: serum creatinine 2-2.9 times baseline, Urine output < 0.5 mL/kg/h for 12 h.
2. Appropriate blood volume and central venous pressure (CVP) ≥ 6 mmHg; and.
3. Urine output ≤ 0.5 ml/kg/h for 6 h.

Exclusion criteria:

1. Age < 18 years;
2. Indications for emergency CRRT: hyperkalemia, potassium of blood ≥ 6.5 mmol/L; metabolic acidosis, PH ≤ 7.15; acute pulmonary edema due to fluid overload; developed uremia-related complications, such as pericarditis, bleeding, etc.;
3. Chronic kidney disease or having received renal replacement therapy 30 days before inclusion;
4. Presence of postrenal obstruction factors;
5. Evidence of volume depletion at the time of furosemide administration.

### Study design

In this single-group quasi-experimental observational design, with no randomization or blinding since there were no comparison or control groups. All eligible patients who met the inclusion criteria during the study period received the intervention.

Screening involves reviewing patients’ medical records, laboratory test results, and clinical assessments. Informed consent was obtained from all participants or their legal representatives before study enrollment.

The study was started after obtaining the Research Ethics Committee’s approval **(N-381-2024)** and conducted for 6 months, including patient recruitment, data collection, and analysis.

### Methodology

This is a prospective quasi-experimental study; it included 41 critically ill patients with AKI stage 1 and 2 admitted to the ICU and meeting the AKI diagnostic criteria for Kidney Disease Improving Global Outcomes (KDIGO) guidelines (stage 1 and 2).

Timing of FST Administration:

Kidney function was assessed using the furosemide stress test immediately after confirming AKI and hypoalbuminemia. This timeline was selected to evaluate kidney function rapidly before potential influences from subsequent clinical interventions occurred.

Dose of furosemide: IV furosemide 1 mg/kg for diuretic-naive patients and 1.5 mg/kg for those with previous diuretic treatment.

The patients were considered responders if the urine output exceeded 200 ml in the 2 h following furosemide injection, and non-responders otherwise.

To standardize conditions during the study:

- Allowed Medications: The research team allowed patients to maintain their necessary medications used for treatment, like vasopressors, antibiotics, and electrolyte supplements.
- Prohibited Medications: Other diuretics, albumin infusion, and nephrotoxic medications like aminoglycosides and NSAIDs need to be stopped for 24-hour intervals before the test.

Fluid intake was adjusted according to their current hydration levels when giving standard maintenance fluids. A fluid bolus was avoided within six hours before the FST to ensure that fluid status does not interfere with diuretic responsiveness.

Patients were managed according to standard critical care protocols, and were continuously monitored during the furosemide stress test for:

- Hemodynamic parameters: The team monitored blood pressure levels together with heart rate and measured central venous pressure whenever possible.
- Urine output: Our team tracked urine output every hour during the two-hour period after the patient received furosemide.
- Electrolytes: we measured sodium, potassium, and bicarbonate levels in the blood at the test start and analyzed them regularly afterward to find any problems during and after testing.
- Signs of adverse effects: The team checked for changes in fluid balance as well as low blood pressure and abnormal electrolytes that could need medical attention.

Identified patients who received nephrotoxic drugs, vasopressors or have diabetes, on mechanical ventilation or not, sepsis (by qSOFA), or hypoalbuminemia.

Suspicion of infection and sepsis were made using the following data extracted from hospital records: blood pressure, heart rate, body temperature, respiratory rate, and level of consciousness. Demographic and laboratory variables were recorded for all patients. We calculated quick sequential organ failure assessment (qSOFA)for each patient. The score ranges from 0 to three with one point allocated for each clinical sign: systolic blood pressure < 100 mmHg respiratory rate > 22/minutes and altered mental status from baseline. A score of equal or more than two indicates more severity with increased ICU length of stay and mortality.

### Statistical analysis

A convenient sample of 41 acute kidney injury (AKI) patients eligible for the furosemide stress test will be included in this study. The sample size was calculated based on the primary outcome measure, which is a comparison of urine output before treatment and after 2 h. So., based on prior data from (McMahon, B. A., Chawla, L. S., & Patel, N. R. 2021) [11] and using the G power program T test family with paired comparison; 41 patients were calculated with the following statistical assumption effect size d 0.466, Power 80%, 0.05 significant level and dropout rate 10%. Analysis of data was done by an IBM computer using SPSS (statistical program for social science version 23) as follows:

- Description of quantitative variables as median and IQR (interquartile range) according to Shapiro’s test of normality.
- Description of qualitative variables as frequency and percentage.
- Fisher’s exact test was used to compare qualitative variables between groups.
- Mann-Whitney test was used instead of the unpaired t-test in non-parametric data.
- Spearman correlation to test for bivariate correlation between variables.
- ROC curve analysis (ROC) to assess the discriminant ability of albumin and SOFA in frusemide response determination.
- P value ≤ 0.05 significant.

## Results

### Patient baseline characteristics

41 patients with hypoalbuminemia and AKI were enrolled (Table 1). The median age was 54.63 ± 10.32 years, with 27 males (65.9%) and 14 females (34.10%).

**Fig. 1:**
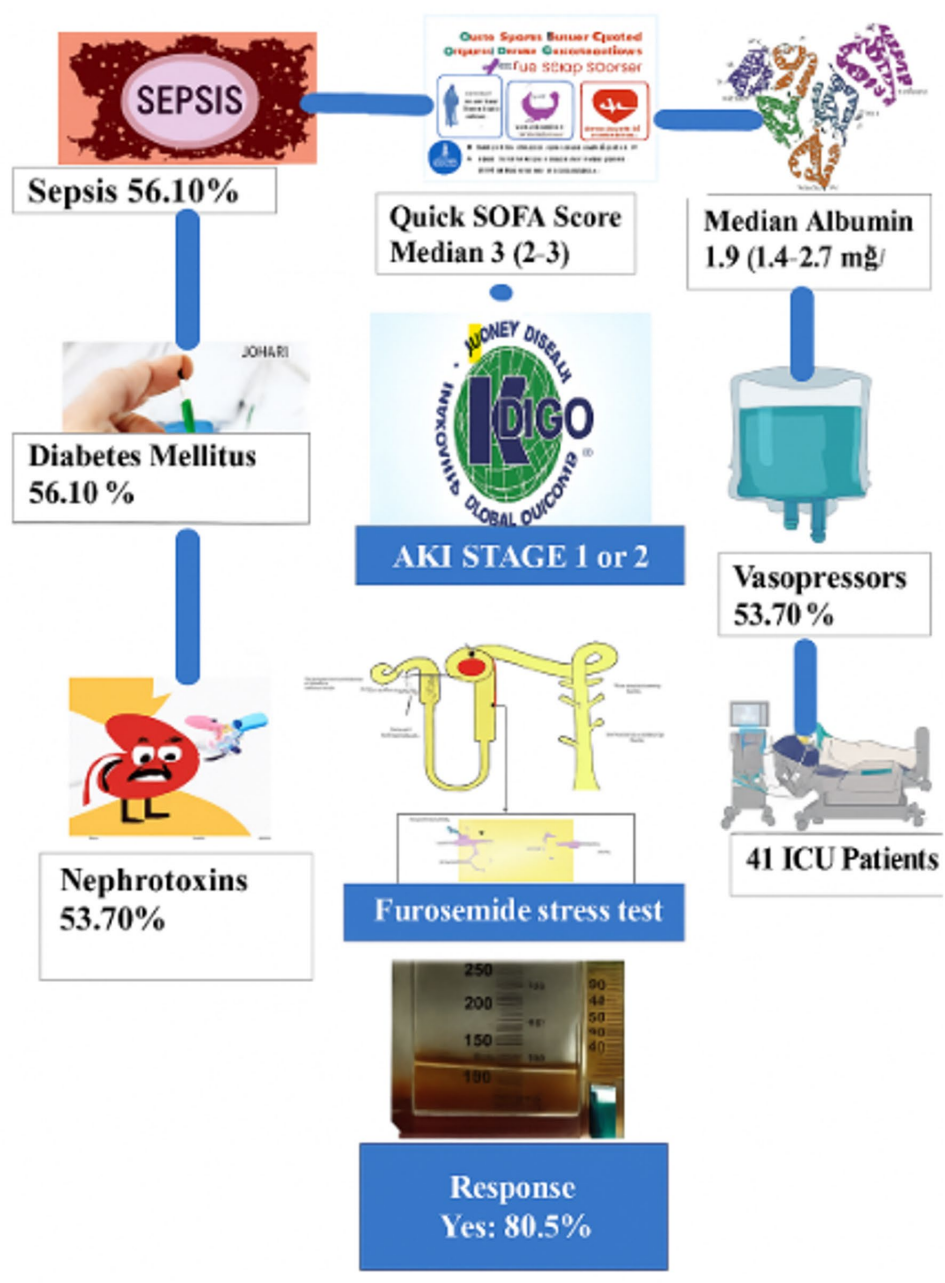
Patient flow diagram

Key baseline characteristics included (Fig. 1):

- AKI staging: 31 patients (75.60%) with stage 1 and 10 patients (24.40%) with stage 2.
- Median serum albumin: 1.9 g/dL (IQR: 1.4–2.7).
- Median Quick SOFA score: 3 (IQR: 2–3).
- Median GCS: 9 (IQR: 8–12).

**Table 1:** Baseline characteristics

Patient characteristics		Frequency	%
Type AKI	Sepsis	23	56.10%
	No Sepsis	18	43.90%
Sex	Male	27	65.90%
	Female	14	34.10%
Diabetes	Yes	23	56.10%
	No	18	43.90%
KDIGO class	Stage1	31	75.60%
	Stage 2	10	24.40%
Nephrotoxic drugs	Yes	22	53.70%
	No	19	46.30%
Vasopressors	Yes	22	53.70%
	No	19	46.30%
Mechanical ventilation	Yes	26	63.40%
	No	15	36.60%
**Patient characteristics**	**Median (IQR)**	**Range**	
Age	54.63 ± 10.32	30–77	
Serum albumin	1.9(1.4–2.7)	1.1–3.3	
Dose of frusemide in mg	80(80–100)	60–120	
SOFA	2(2–3)	1–4	
Quick SOFA score	3(2–3)	1–3	
GCS	9(8–12)	6–14	

AKI = acute kidney injury, KDIGO = Kidney Disease Improving Global Outcomes

GCS = Glasgow coma scale, SOFA = Sequential Organ Failure Assessment

**Comorbidities and clinical conditions included (Fig. ****1****)**:

- Diabetes: 23 patients (56.10%).
- Sepsis as AKI cause: 23 patients (56.10%).
- On nephrotoxic medications: 22 patients (53.70%).
- Requiring vasopressors: 22 patients (53.70%).
- On mechanical ventilation: 26 patients (63.40%).

### Primary outcome: response to FST

Of the 41 hypoalbuminemic patients, 33 patients (80.5%) responded to FST without prior albumin infusion. Response rates varied significantly by (Table 2):

a) Albumin levels:

- Responders: median albumin 2.0 g/dL (IQR: 1–3).
- Non-responders: median albumin 1.0 g/dL (IQR: 1–2).
- (*P* = 0.002)

b) AKI stage:

- Stage 1 patients showed significantly better response (*P* = 0.013).

c) Factors Associated with FST Response:

Multivariable logistic regression analysis was conducted to identify clinical factors associated with response to the Furosemide Stress Test among critically ill hypoalbuminemic patients with AKI stages 1 and 2. The model included baseline serum albumin level, illness severity scores (SOFA, quick SOFA), AKI stage, presence of sepsis, diabetes mellitus, vasopressor use, exposure to nephrotoxic drugs, mechanical ventilation, and patient age.

The analysis revealed that:

- Higher baseline serum albumin was independently associated with a positive FST response (OR: 2.15, 95% CI: 1.30–3.55, *p* = 0.003),
- Lower SOFA scores were also significantly associated with positive response (OR: 0.68, 95% CI: 0.50–0.92, *p* = 0.012). Other variables, including quick SOFA, AKI stage, sepsis, diabetes mellitus, vasopressor use, nephrotoxic drug exposure, mechanical ventilation, and age did not show statistically significant associations with FST response in the adjusted model.

These findings suggest that, beyond the severity of tubular injury, factors such as hypoalbuminemia and overall illness severity significantly influence responsiveness to furosemide among this patient population.

### Predictive analysis

**Fig. 2:**
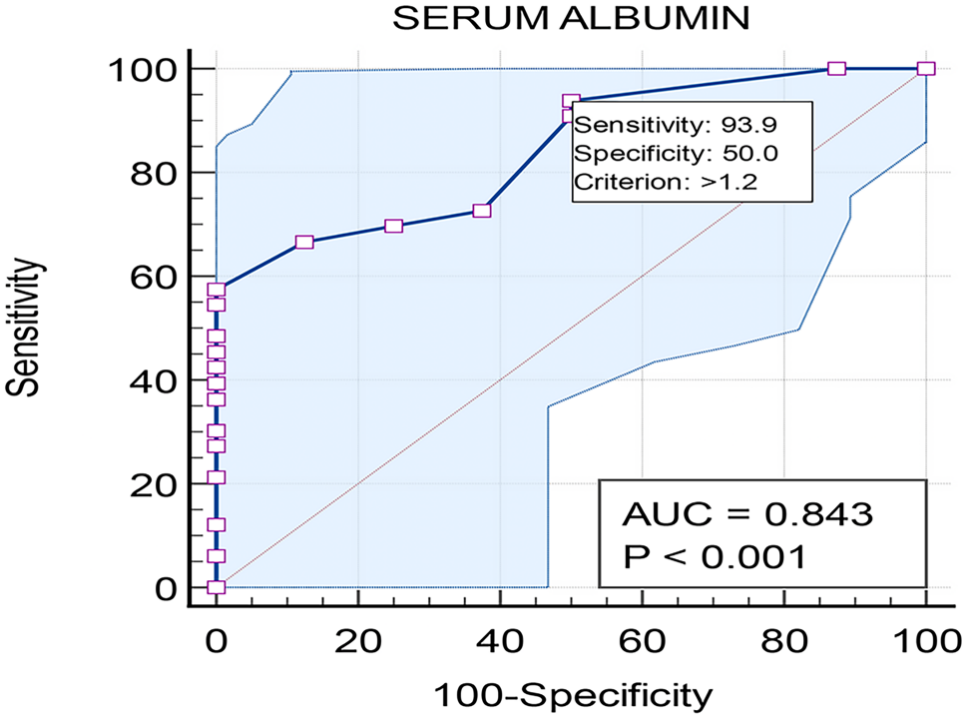
ROC curve analysis for albumin

**Fig. 3:**
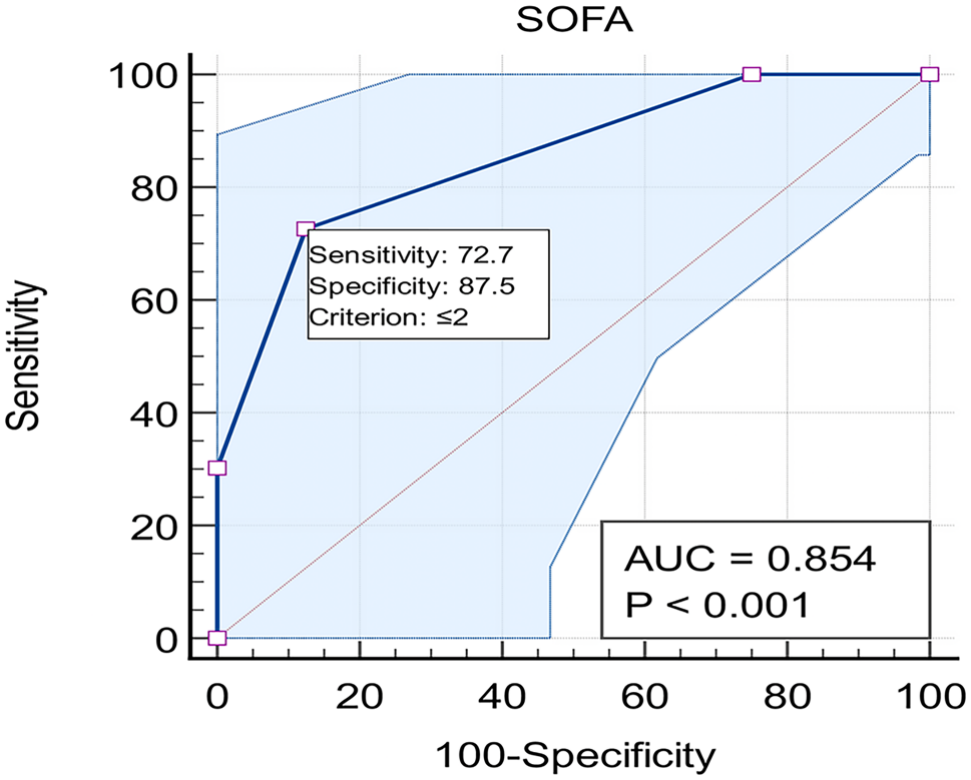
ROC curve analysis for SOFA

ROC curve analysis revealed:

a) Serum Albumin as a predictor (Fig. 2):

- AUC: 0.843.
- Optimal cut-off: >1.2 g/dL.
- Sensitivity: 93.94% (95% CI: 79.8–99.3).
- Specificity: 50% (95% CI: 15.7–84.3).

b) SOFA score as a predictor (Fig. 3):

- AUC: 0.854.
- Optimal cut-off: ≤2.
- Sensitivity: 72.73% (95% CI: 54.5–86.7).
- Specificity: 87.5% (95% CI: 47.3–99.7)”.

**Table 2:** Factors associated with FST response

Response to FST				
Factors		No	Yes	*P* value
		Frequency (%)	Frequency (%)	
Type AKI	Sepsis	7 (87.5)	16 (48.5)	
	No Sepsis	1 (12.5)	17 (51.5)	0.059
Sex	Male	5 (62.5)	22 (66.7)	1
	Female	3 (37.5)	11 (33.3)	
Diabetes	Yes	7 (87.5)	16 (48.5)	
	No	1 (12.5)	17 (51.5)	0.059
KDIGO class	Stage1	3 (37.5)	28 (84.8)	0.013
	Stage 2	5 (62.5)	5 (15.2)	
Nephrotoxic drugs	Yes	4 (50)	18 (54.5)	
	No	4 (50)	15 (45.5)	1
Vasopressors	Yes	7 (87.5	15 (45.5)	
	No	1 (12.5)	18 (54.5)	0.05
Mechanical ventilation	Yes	8 (100)	18 (54.5)	
	No	0 (0)	15 (45.5)	0.018
*Age	Median (IQR)	59 ± 6	54 ± 11	0.213
	Range	48–69	30–77	
^SOFA	Median (IQR)	3(3–4)	2(1–3)	0.001
	Range	2–4	1–3	
quick SOFA score	Median (IQR)	3(3–3)	2(1–3)	0.024
	Range	3–3	1–3	
^GCS	Median (IQR)	8(7–8)	10(8–13)	0.005
	Range	6–12	6–14	
^Serum albumin	Median (IQR)	1(1–2)	2(1–3)	0.002
	Range	1–2	1–3	
Dose of furosemide in mg	Median (IQR)	100(100–110)	80(80–100)	0.12
	Range	60–120	60–120	

Fisher Exact ^Mann Whitney U test * Independent t test p value ≤ 0.05

FST = furosemide stress test, AKI = acute kidney injury, KDIGO = Kidney Disease Improving Global Outcomes

GCS = Glasgow coma scale, SOFA = Sequential Organ Failure Assessment

## Discussion

Our study demonstrated that 80.5% of AKI patients with hypoalbuminemia responded to FST without prior albumin infusion. We identified critical factors affecting response, including baseline albumin levels (cutoff > 1.2 g/dL), AKI stage, and the absence of vasopressor support. These findings challenge the conventional practice of routine albumin administration before furosemide in hypoalbuminemic patients.

While previous studies have suggested that hypoalbuminemia may impair furosemide’s efficacy due to reduced protein binding and decreased tubular secretion (Smith et al., 2015; Johnson et al., 2017) [12, 13]. our findings indicate that this may not be clinically significant above certain albumin thresholds. This aligns with Ahmed et al.‘s (2019) work showing effective diuretic response without albumin supplementation in mild-to-moderate hypoalbuminemia but contrasts with Brown’s (2021) findings in severe hypoalbuminemia [14, 15]. Liang Xu et al.‘s (2025) work supports our findings on the effectiveness of FST in critically ill patients with AKI, they found that urine output > 188 mL in the first 2 h after FST predicts successful discontinuation of CRRT [16].

Our results confirm that while albumin below a certain threshold compromises the FST response, worsening to KDIGO stage 2, defined by increased tubular injury but further impairs diuresis, most likely because of altered furosemide delivery and action at the nephron. Both decreased serum albumin and more severe AKI, therefore, act synergistically to impair FST responsiveness.

The limited reports specifically investigating furosemide pharmacokinetics in AKI validate our findings. Studies referenced under PMID: 30,225,802 and PMID: 27,183,025 have proven that with more serious AKI (low creatinine clearance), furosemide’s diuretic and natriuretic effects are reduced, even at higher doses. Tripodi et al. (PMID: 30225802) demonstrated that urine volume produced per milligram of furosemide was lower in patients with more severe AKI, reflecting poor tubular secretion and reduced delivery of furosemide to the site of action [17]. Concomitantly, Ehrenpreis et al.‘s (PMID: 27183025) article understands that furosemide dose and existing renal function (as determined by creatinine clearance) both directly affect diuretic response in a fashion independent of serum albumin level [18]. This result is of the most significance to clinicians who use the FST for risk stratification and decision-making in hypoalbuminemic AKI patients.

The observed response pattern may be explained by several mechanisms:

- Sufficient free drug fraction reaching the tubular secretion sites even with lower albumin levels.
- Compensatory mechanisms in early AKI maintaining tubular function.
- The role of critical illness severity (as evidenced by our SOFA score findings) in modulating diuretic response [19].

Clinical Implications:

Our findings have several practical implications:

- FST without albumin supplementation may be appropriate for patients with albumin levels > 1.2 g/dL.
- Early AKI (KDIGO stage 1) appears more responsive to FST.
- The presence of mechanical ventilation and vasopressor support may predict poor response.
- A SOFA score ≤ 2 might help identify patients more likely to respond to FST.

### The study bias and limitations

Our study has some bias that need to be emphasized. The sample size (41 patients) was relatively small, limiting the findings’ statistical power and generalizability. Moreover, being a quasi-experimental study rather than a randomized controlled trial introduces some potential selection bias.

On the other hand, no control group received albumin supplementation for direct comparison. Lastly, the study is a single-center study, limiting external validity. Our patients also have a wide age range (18–80 years), introducing some heterogeneity in the study population with a high prevalence of comorbidities (diabetes, nephrotoxic medications, sepsis) that could confound our results.

In addition, we only included KDIGO stages 1 and 2 AKI, excluding more severe cases and without long-term follow-up. No mortality data is provided and no analysis of secondary outcomes or complications. We need to assess the progression of chronic kidney disease in future studies with a cost-benefit analysis.

Further research should focus on Larger multicenter trials to validate our findings, Cost-effectiveness analysis of selective versus routine albumin supplementation, Investigation of long-term outcomes, Identification of additional predictive biomarkers, and Development of personalized approaches based on patient characteristics [20].

## Conclusion

The study suggests that FST without albumin supplementation can be effective in selected AKI patients with hypoalbuminemia, particularly those with higher baseline albumin levels and lower illness severity scores. This finding could lead to more cost-effective and targeted use of albumin supplementation in clinical practice.

## Data Availability

All data generated or analysed during this study are provided within the manuscript [and its supplementary information file].

## Abbreviations

AKI: Acute Kidney Injury
AUC: Area under the curve
CKD: Chronic kidney disease
CRRT: Continuous renal replacement therapy
CVP: Central venous pressure
GCS: Glasgow coma scale
ICU: Intensive care unit
IQR: Interquartile range
KDIGO: Kidney Disease Improving Global Outcomes
RRT: Renal replacement therapy
SOFA: Sequential Organ Failure Assessment
SPSS: Statistical program for social science
